# Third-generation inhibitors targeting *EGFR* T790M mutation in advanced non-small cell lung cancer

**DOI:** 10.1186/s13045-016-0268-z

**Published:** 2016-04-12

**Authors:** Shuhang Wang, Shundong Cang, Delong Liu

**Affiliations:** The Key Laboratory of Carcinogenesis and Translational Research (Ministry of Education), Peking University Cancer Hospital, Beijing, China; Department of Oncology, People’s Hospital of Henan Province, Zhengzhou University, Zhengzhou, China; Department of Oncology, Henan Cancer Hospital, Zhengzhou University, Zhengzhou, China; Henan Cancer Hospital, Zhengzhou University, Zhengzhou, China

## Abstract

The tyrosine kinase inhibitors (TKI) against epidermal growth factor receptor (EGFR) are widely used in patients with non-small cell lung cancer (NSCLC). However, *EGFR* T790M mutation leads to resistance to most clinically available EGFR TKIs. Third-generation EGFR TKIs against the T790M mutation have been in active clinical development. These agents include osimertinib, rociletinib, HM61713, ASP8273, EGF816, and PF-06747775*.* Osimertinib and rociletinib have shown clinical efficacy in phase I/II trials in patients who had acquired resistance to first- or second-generation TKIs. Osimertinib (AZD9291, TAGRISSO) was recently approved by FDA for metastatic *EGFR* T790M mutation-positive NSCLC. HM61713, ASP8237, EGF816, and PF-06747775 are still in early clinical development. This article reviews the emerging data regarding third-generation agents against EGFR T790M mutation in the treatment of patients with advanced NSCLC.

## Background

**Fig. 1:**
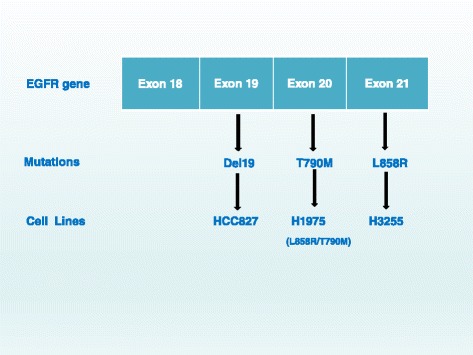
Common mutations of the epidermal growth factor receptor (EGFR) that are targets for tyrosine kinase inhibitors. Exons 18 to 21 of EGFR gene are common sites for mutations. The most common mutations that are targets of EGFR TKIs are shown. As an example, three mutation-harboring lung cancer cell lines commonly used to characterize EGFR TKIs are also shown. *Del19:* exon 19 deletion

Non-small cell lung cancer (NSCLC) accounts for approximately 85 % of all lung cancers. The 5-year survival rate in advanced NSCLC patients is less than 5 %. The activating mutations of epidermal growth factor receptor (EGFR) occur in approximately 10–15 % of NSCLC cases in Caucasian patients and approximately 30–40 % in East Asian patients [1, 2]. The first- and second-generation EGFR tyrosine kinase inhibitors (TKI), erlotinib, gefitinib, and afatinib, have been widely used for these advanced NSCLC patients [3–5]. However, acquired resistance to these inhibitors frequently develops after a median of 9 to 13 months [5–11]. The common acquired *EGFR* mutations with clinical implications are exon 19 deletions (del19), L858R mutation, and the T790M mutation (Fig. 1) [2]. Cell lines harboring these mutations have been used for screening novel agents targeting these mutations [12]. The *EGFR* T790M mutation was present in approximately 50 to 60 % of resistant cases [13, 14]. The median survival is less than 2 years after the emergence of T790M mutation [13]. Recently, the third-generation EGFR inhibitors, AZD9291 (osimertinib, mereletinib), CO-1686 (rociletinib), HM61713 (BI 1482694), ASP8273, EGF816, and PF-06747775, have emerged as potential therapeutics to block the growth of *EGFR* T790M-positive tumors [15–17]. More importantly, unlike the first- and second-generation EGFR TKIs, the third-generation TKIs have a significantly increased potency for *EGFR* mutants than for wild-type *EGFR*.

## AZD9291 (osimertinib, mereletinib, tagrisso)

AZD9291 is structurally different from the first- and second-generation EGFR TKIs. This compound is an irreversible mutant-selective EGFR TKI (exon 19 deletion *EGFR* IC_50_ = 12.92 nM, L858R/T790M *EGFR* IC_50_ = 11.44 nM, wild-type EGFR IC_50_ = 493.8 nM) [15]. It is the only approved EGFR TKI currently indicated for patients with metastatic *EGFR* T790M mutation-positive NSCLC [18].

A phase I dose escalation study of AZD9291 (AURA) was done in patients with advanced *EGFR*-mutated NSCLC who had disease progression from the first-generation of EGFR TKI treatment. Patients received doses ranging from 20 to 240 mg/day. These cohorts were not preselected according to *T790M* status. Five expansion cohorts were stratified according to *T790M* status (*T790M*-positive or -negative). Thirty-one patients were enrolled in the escalation cohort and 222 additional patients in the five expansion cohorts. The objective response rate (ORR) was 51 % in the whole population (95 % CI 45–58 %). Among 127 evaluable patients with *EGFR* T790M mutation, ORR was 67 % (95 % CI 52–70 %). The response rates were similarly high across the five tested dose levels. For patients without *EGFR* T790M, the ORR was 21 % (95 % CI 12–34 %) [19]. The median progression-free survival (PFS) was longer in *EGFR* T790M-positive patients (9.6 months; 95 % CI 8.3 to not reached) than that in *EGFR* T790M-negative patients (2.8 months; 95 % CI 2.1–4.3). The most common adverse effects (AE) were rash, diarrhea, nausea, and poor appetite. There were no dose-limiting toxicities (DLTs) at any dose level. Maximum tolerated dose (MTD) was not reached. At higher dose levels of 160 and 240 mg, an increase in the incidence and severity of adverse events (rash, dry skin, and diarrhea, etc.) was observed. This was thought to be associated with inhibition of non-mutant *EGFR*. Therefore, 80 mg daily was recommended to be used for further clinical trials [19]. This study also suggested that *EGFR* T790M is not only a prognostic but also a predictive biomarker.

AZD9291 has been examined in the first-line treatment in an expansion cohort from AURA trial, doses of 80 or 160 mg/day were administered to 60 treatment-naïve patients with *EGFR*-mutated advanced NSCLC at the time of report [20]. The median age of the patients was 63.5, stable brain metastasis was allowed. *EGFR* mutation subtypes included *EGFR* exon 19 deletion (37 %), *EGFR* exon 21 L858R (40 %), other *EGFR* sensitizing mutations (3 %), and T790M in 8 % of patients. ORR at the cutoff date was 70 % (95 % CI 57–81). A third of the patients had grade ≥3 adverse events, mainly including skin rash and diarrhea. These results appeared to be promising but clearly preliminary.

An ongoing first-line phase III trial is comparing the efficacy and safety of AZD9291 (80 mg/day) in combination with gefitinib or erlotinib in patients with common *EGFR* mutations. The primary end point is PFS, and the secondary end points include assessment of PFS by pretreatment T790M mutation status and by *EGFR* mutation subtype (exon 19 deletion or L858R) detected in circulating tumor DNA. Patients were allowed to cross over to AZD9291 after disease progression in the control arm (Table 1).

**Table 1:** Ongoing clinical trials of osimertinib (AZD9291, TAGRISSO)

Phase	Study population	NCT no.
Phase Ib	EGFR mutated advanced NSCLC progressed following therapy with an EGFR TKI	02143466
Phase I	EGFR mutated advanced NSCLC progressed following therapy with an EGFR TKI and standard therapy	02157883
Phase I	EGFR mutated advanced NSCLC progressed following therapy with an EGFR TKI	02317016
Phase III (ADAURA)	EGFR mutated stage IB–IIIA NSCLC following complete resection with or without adjuvant chemotherapy	02511106
Phase I	EGFR mutated advanced NSCLC progressed following therapy with an EGFR TKI	02197234
Phase III	EGFR mutated advanced NSCLC	02296125
Phase III	EGFR T790M mutation-positive NSCLC progressed following therapy with an EGFR TKI	02474355
Phase III	EGFR T790M mutation-positive NSCLC progressed following therapy with an EGFR TKI	02151981
Phase I	EGFR mutated advanced NSCLC progressed following therapy with an EGFR TKI	02503722
Phase I	EGFR mutated advanced NSCLC progressed following therapy with an EGFR TKI	02496663
Phase I/II	EGFR mutated advanced NSCLC progressed following therapy with an EGFR TKI	01802632
Phase I	EGFR mutated advanced NSCLC	02228369
Phase II	EGFR T790M mutation-positive NSCLC progressed following therapy with an EGFR TKI	02094261
Phase II	EGFR T790M mutation-positive NSCLC progressed following therapy with an EGFR TKI	02442349
Phase Ib	EGFR mutated advanced NSCLC progressed following therapy with an EGFR TKI	02520778
Phase IIa	Stage IIIB–IV locally advanced or metastatic NSCLC	02179671
Phase I	Chinese patients with EGFR mutated advanced NSCLC progressed following therapy with an EGFR TKI	02529995
Phase II	EGFR mutated advanced NSCLC progressed following therapy with an EGFR TKI	02504346

EGFR: epidermal growth factor receptor, NSCLC: non-small cell lung cancer, TKI: tyrosine kinase inhibitor

Using EGFR mutant cell lines, investigators discovered additional resistance mechanisms, such as NRAS and KRAS mutations and overexpression. A combination of AZD9291 with the MEK inhibitor, selumetinib, was shown to cause regression of AZD9291-resistant tumors in an *EGFR*m/T790M transgenic model [21]. Multiple ongoing trials of AZD9291 in combination with other novel agents are listed in Table 1. These agents include selumetinib (MEK inhibitor), necitumumab (EGFR antibody), navitoclax (inhibitor of Bcl-xL, Bcl-2, and Bcl-w) [22], and AZD6094 (MET inhibitor).

## Rociletinib (CO-1686)

Rociletinib is another novel, oral, irreversible mutant-selective inhibitor of commonly mutated forms of *EGFR* (exon 19 deletion, L858R, and T790M). Preclinical studies have shown that rociletinib has minimal activity against wild-type EGFR [16]. In xenograft and transgenic models of NSCLC with *EGFR* mutations including T790M, rociletinib resulted in durable tumor shrinkage [16].

A phase I/II study of rociletinib was done in patients with *EGFR*-mutated NSCLC with acquired resistance to first- or second-generation EGFR inhibitors [23]. In the phase II part of the study, patients with NSCLC positive for *EGFR* T790M received rociletinib at doses of 500, 625, or 750 mg twice daily. At the time of report, 130 patients were enrolled. MTD was not identified. One common DLT was hyperglycemia. Among the 46 patients with T790M-positive disease who could be evaluated, the ORR was 59 % (95 % CI 45 to 73). For the 17 patients with T790M-negative disease, the ORR was 29 % (95 % CI 8 to 51). Therefore, rociletinib was active in NSCLC patients with *EGFR* T790M mutation.

The confirmatory phase II trial of second-line rociletinib (625 mg twice a day) for advanced *EGFR*-mutated NSCLC that progressed after previous EGFR TKI therapy is ongoing (TIGER-2; NCT02147990). A randomized phase II study of first-line rociletinib versus erlotinib monotherapy for *EGFR*-mutated advanced NSCLC already started recruiting. The phase III trial, TIGER-3, is another open-label, multicenter, randomized study of rociletinib monotherapy versus single-agent cytotoxic chemotherapy in NSCLC patients with mutant *EGFR* after failure of at least one previous EGFR TKI and platinum-doublet chemotherapy. The ongoing trials of rociletinib are summarized in Table 2.

**Table 2:** Ongoing clinical trials of rociletinib (CO-1686)

Phase	Study population	NCT no.
Phase I/II	EGFR mutated advanced NSCLC	02580708
Phase I/II	EGFR mutated advanced NSCLC	02630186
Phase II/III (TIGER-1)	Untreated EGFR mutated advanced NSCLC	02186301
Phase II (TIGER-2)	EGFR mutated NSCLC	02147990
Phase III (TIGER-3)	EGFR mutated advanced NSCLC after failure of ≥1 previous EGFR TKI and platinum-doublet chemotherapy	02322281

EGFR: epidermal growth factor receptor, NSCLC: non-small cell lung cancer, TKI: tyrosine kinase inhibitor

## HM61713 (BI 1482694)

HM61713 is an irreversible kinase inhibitor and covalently binds to a cysteine residue near the kinase domain of mutant EGFR. HM61713 has a half-life of over 24 h for EGFR inhibition [24]. HM61713 caused potent inhibition in cell lines H1975 (L858R and T790M) and HCC827 (exon 19 deletion) (Fig. 1). HM61713 has a low potency for NSCLC cell line H358 harboring wild-type EGFR (GI_50_ of 2225 nM). In the in vivo studies of xenograft models with grafts of H1975 and HCC827, HM61713 was active against the tumors without showing any side effects [24].

In the ongoing phase I/II study of HM61713 in patients with advanced NSCLC who had failed previous EGFR TKIs (NCT01588145), *EGFR* mutation-positive patients received doses ranging from 75 to 1200 mg/day [17]. In the phase II expansion part of the study, 800 mg QD was the dose given to patients with centrally confirmed T790M-positive NSCLC. In the latest update, 173 patients were enrolled, including 55 in the phase I and 118 in the phase II cohorts. Eight hundred milligrams once daily was the MTD. DLTs mainly involved GI symptoms and elevation of aspartate aminotransferase, alanine aminotransferase, amylase, and lipase. The ORR was 58.8 % in the 34 patients who received HM61713 with a dose more than 650 mg. In addition, ten patients had unconfirmed partial responses, and 13 achieved disease stabilization [17]. Therefore, HM61713 represents another promising agent for patients with T790M-positive NSCLC.

## ASP8273

ASP8273 is another small molecule, irreversible TKI inhibitor that inhibits the kinase activity of *EGFR* mutations including T790M, with limited activity against *EGFR* wild-type (WT) NSCLC. In the in vitro enzymatic and cell-based assays, ASP8273 were evaluated against *EGFR* mutants (L858R, exon 19 deletion, L858R/T790M, and del19/T790M) and WT *EGFR* [25]. ASP8273 was found by mass spectrometry to covalently bind to a mutant EGFR (L858R/T790M) via cysteine residue 797 in the kinase domain of EGFR with long-lasting inhibition of EGFR phosphorylation for 24 h. In the NSCLC cell lines harboring the above EGFR mutations, ASP8273 had IC_50_ values of 8–33 nM toward EGFR mutants, more potently than that of WT EGFR (IC_50_ value of 230 nM). In mouse xenograft models, ASP8273 induced complete regression of the tumors after 14 days of treatment [25]. ASP8273 was further shown to suppress the signaling pathway through ERK and Akt. ASP8273 even showed activity in mutant EGFR cell line which is resistant to other EGFR TKIs including AZD9291 and CO-1686 [26]. Therefore, ASP8273 represents a unique agent active in NSCLC with *EGFR* T790M mutation.

ASP8273 was evaluated in an open-label phase I/II study for safety and efficacy [27]. As of late 2014, 30 Japanese patients were enrolled in the phase I cohorts across seven dose levels (25–600 mg), and 15 patients were enrolled in the expansion cohorts across four dose levels (100–400 mg). *EGFR* T790M mutation was positive in 49 %. There were 13 % negative for T790M and 38 % unknown. The most common AEs were GI toxicity and thrombocytopenia (31 %). DLTs were reported at higher dose levels (400–600 mg). MTD was established as 400 mg. PR was achieved in 50 % (18/36) of all evaluable patients and 80 % (12/15) in those with T790M. From this study, 300 mg once daily was chosen as the recommended phase II dose (RP2D) [27].

In a separate report of a phase I/II study with cutoff date of January 2015, 24 patients were enrolled across six dose escalation cohorts (25–400 mg once daily) and 11 patients were enrolled in two expansion cohorts (100–200 mg once daily) [28]. All patients had failed prior erlotinib treatment. The most common treatment-related AEs were mild gastrointestinal toxicities. DLTs included hyponatremia and anorexia (one case each). The ORR among the evaluable patients was 28 % (7/25) at the cutoff date. In patients with T790M mutation, the ORR was 25 % (3/12). The MTD was anticipated to be 400 mg and the RP2D to be 300 mg. More ongoing studies are listed in Table 3.

**Table 3:** Ongoing clinical trials of ASP8273

Phase	Study population	NCT no.
Phase I	NSCLC patients who have EGFR mutations and received prior treatment with EGFR TKI	02113813
Phase I/II	NSCLC with EGFR mutation and had progressive disease after previous treatment with EGFR TKIs	02192697
Phase II	NSCLC with EGFR mutation and TKI naïve patients	02500927
Phase III	Stage IIIB/IV NSCLC with EGFR mutations	02588261

EGFR: epidermal growth factor receptor, NSCLC: non-small cell lung cancer, TKI: tyrosine kinase inhibitor

## EGF816

EGF816 is another third-generation covalent EGFR inhibitor that has potent inhibitory activity against activating (L858R, del19) and resistant T790M mutants with low IC_50_ in various cellular assays [12]. In mouse xenograft models, EGF816 was better than earlier generation EGFR inhibitors. EGF816 target profiles suggest that it represents an alternative and better therapy option against T790M mutations [12, 29–31].

A phase I multicenter, dose escalation study of EGF816 enrolled NSCLC patients with confirmed T790M status. The starting dose was 75 mg daily. The doses were escalated according to an adaptive Bayesian logistic regression model. At the cutoff date of 26 January 2015, 57 patients were treated across six cohorts (75, 150, 225, 300, and 350 mg for capsules; 225 mg for tablets). Diarrhea, stomatitis, rash, and pruritus were the most common AEs. ORR was 54.5 % in 22 evaluable patients. The ongoing study is to determine the MTD and RP2D [32]. More ongoing studies are listed in Table 4.

**Table 4:** Ongoing clinical trials of EGF816

Phase	Study population	NCT no.
Phase I/II	Patients with EGFR mutated solid malignancies	02108964
Phase Ib/II	Patients with EGFR mutated NSCLC	02335944
Phase II	Patients with EGFR mutated and cMET-positive NSCLC	02323126

EGFR: epidermal growth factor receptor, NSCLC: non-small cell lung cancer, TKI: tyrosine kinase inhibitor

## PF-06747775

PF-06747775 is another small molecule inhibitor of EGFR T790M. This molecule is being studied in phase I/II clinical trial (NCT02349633) in advanced NSCLC patients with EGFR mutations (del 19 or L858R ± T790M). The agent will be administered as continuous daily dosing in 21-day cycles. The starting dose of PF-06747775 will be 25 mg PO daily.

## Resistance to third-generation EGFR inhibitors

New mutations are emerging that mediate resistance to third-generation EGFR TKIs [33–37]. Among these, C797S mutation was found to be the most common mechanism responsible for resistance to AZD9291 [33, 35, 36]. C797S was also reported in one case that led to resistance to HM61713 [34]. The C797S mutation was reported to arise after approximately 6–17 months of treatment in patients with T790M mutations [33–36]. Additional mutations and mechanisms of resistance to EGFR TKIs clearly exist since resistance to AZD9291 was reported in patients who became negative for T790M mutation and had no C797S mutation [35].

## Conclusions

Clinical trials are being done on the third-generation EGFR TKIs, osimertinib, rociletinib, HM61713, ASP8273, EGF816, and PF-06747775 that are effective for T790M *EGFR* mutants*.* Osimertinib (AZD9291, TAGRISSO) was recently approved by FDA for metastatic *EGFR* T790M mutation-positive NSCLC. The other five inhibitors are still in clinical development. Novel agents are needed to conquer the C797S tertiary EGFR mutation. Since ALK inhibitors and immune check point blockers are also widely used for treatment of NSCLC [8, 38–46], combination and sequential therapies with these agents may improve outcome in patients with advanced NSCLC.
